# Integrative analysis of methylomic and transcriptomic data in fetal sheep muscle tissues in response to maternal diet during pregnancy

**DOI:** 10.1186/s12864-018-4509-0

**Published:** 2018-02-06

**Authors:** Hadjer Namous, Francisco Peñagaricano, Marcello Del Corvo, Emanuele Capra, David L. Thomas, Alessandra Stella, John L. Williams, Paolo Ajmone Marsan, Hasan Khatib

**Affiliations:** 1 Department of Animal Sciences, University of Wisconsin, 1675 Observatory Drive, Madison, WI 53706 USA; 2 Department of Animal Sciences, University of Florida Genetics Institute, University of Florida, Florida, USA; 3 Institute of Zootechnics and PRONUTRIGEN Research Center, Faculty of Agricultural, Food and Environmental Sciences, Università Cattolica del S. Cuore, Piacenza, Italy; 4 Istituto di Biologia e Biotecnologia Agraria, Consiglio Nazionale delle Ricerche, Lodi, Italy; 5 Davies Research Centre, School of Animal and Veterinary Sciences, University of Adelaide, Roseworthy, Australia

**Keywords:** Maternal diet, DNA methylation, Differentially-methylated region, Gene expression

## Abstract

**Background:**

Numerous studies have established a link between maternal diet and the physiological and metabolic phenotypes of their offspring. In previous studies in sheep, we demonstrated that different maternal diets altered the transcriptome of fetal tissues. However, the mechanisms underlying transcriptomic changes are poorly understood. DNA methylation is an epigenetic mark regulating transcription and is largely influenced by dietary components of the one-carbon cycle that generate the methyl group donor, SAM. Therefore, in the present study, we tested whether different maternal diets during pregnancy would alter the DNA methylation and gene expression patterns in fetal tissues.

**Results:**

Pregnant ewes were randomly divided into two groups which received either hay or corn diet from mid-gestation (day 67 ± 5) until day 131 ± 1 when fetuses were collected by necropsy. A total of 1516 fetal longissimus dorsi (LD) tissues were used for DNA methylation analysis and gene expression profiling. Whole genome DNA methylation using methyl-binding domain enrichment analysis revealed 60 differentially methylated regions (DMRs) between hay and corn fetuses with 39 DMRs more highly methylated in the hay fetuses vs. 21 DMRs more highly methylated in the corn fetuses. Three DMRs (LPAR3, PLIN5-PLIN4, and the differential methylation of a novel lincRNA) were validated using bisulfite sequencing. These DMRs were associated with differential gene expression. Additionally, significant DNA methylation differences were found at the single CpG level. Integrative methylome and transcriptome analysis revealed an association between gene expression and inter−/intragenic methylated regions. Furthermore, intragenic DMRs were found to be associated with expression of neighboring genes.

**Conclusions:**

The findings of this study imply that maternal diet from mid- to late-gestation can shape the epigenome and the transcriptome of fetal tissues, and putatively affect phenotypes of the lambs.

**Electronic supplementary material:**

The online version of this article (10.1186/s12864-018-4509-0) contains supplementary material, which is available to authorized users.

## Background

Various studies have demonstrated that maternal diet during the different stages of pregnancy changes the physiological and metabolic phenotypes of the offspring [1], suggesting an adaptation by the fetus known as fetal programming. Changes in fetal epigenetic programming resulting from fetal nutrition during critical stages of development may cause permanent changes in the physiology and metabolism of the individual [2, 3]. The mechanisms by which maternal diet can affect fetal development have been linked with epigenetic marks regulating gene expression without affecting the DNA sequence. These epigenetic marks include DNA methylation and histone modifications [4–6], as well as the activity of microRNAs and long non-coding RNAs [7, 8].

DNA methylation, which is the addition of a methyl group (CH_3_) covalently to cytosine most commonly at the dinucleotide 5´-CpG-3′, is transmitted with high fidelity over many cell generations through mitosis and transgenerationally through meiosis [1, 9]. In mammalian genomes, DNA methylation is fundamental in the control of transcription and in critical processes such as genomic imprinting, and X chromosome inactivation [10–12]. Associations have been established between maternal nutrition and DNA methylation patterns in the fetal genome [6, 13, 14]. DNA methylation is dependent on the one-carbon metabolism pathway, which relies on the availability of nutrients through diet [14]. Nutrients can alter the methylation status of the genome either by direct inhibition of enzymes that catalyze DNA methylation or by changing the availability of substrates necessary for the enzymatic reactions involved [15]. Folic acid, choline, and methionine have been reported to affect the availability of the S-adenosylmethionine (SAM), the methyl group donor used by DNA methyltransferases (DNMTs) [16]. SAM is produced from methionine by L-methionine S-adenosyltransferase. Methionine is provided either by diet or is generated by two pathways involving folate and/or choline. Folate is converted to 5′-methyltetrahydrofolate which is used by methionine synthase to convert homocysteine into methionine, and choline is converted to betaine which is then used by betaine homocysteine methyltransferase to convert homocysteine into methionine [17]. Consequently, deficiency in these nutrients will cause a decrease in the SAM pool leading to a reduction in methylation reactions.

The effects of maternal nutrition on the epigenetic status of their offspring during early development have been demonstrated in several animal species, e.g., the hypomethylation of the long terminal repeat (LTR) region controlling the expression of the agouti gene in mice results in yellow coat color, diabetes, obesity, and decreased survival of offspring [18]. Supplementation of the maternal diet with methyl group donors (folic acid, vitamin B12, methionine, choline and betaine) during pregnancy led to increased methylation of the LTR region, suppression of the agouti gene, and a shift of coat color from yellow to brown [18]. In rats, maternal diet supplemented with folic acid throughout gestation altered global DNA methylation in the brain with no effect on the other organs [19]. In pigs, it was found that sows undernourished throughout gestation (75% of the NRC requirements) gave birth to lighter piglets with decreased expression of *GLUT4* which was coincident with an increase in the methylation of the promoter region of the gene [20]. In a different study of pigs, maternal low-protein diet was associated with high mRNA levels and the hypomethylation of the promoter region of the glucose 6 phosphatase (*G6PC*) gene in the liver of newborn piglets [21]. These effects were predominantly observed in male offspring only suggesting that the maternal diets can have a gender-specific influence [21].

Studies in cattle and sheep have reported alterations of body composition, insulin sensitivity, and growth rate of progeny of dams that were poorly-nourished during certain periods of gestation. Four-month-old male lambs born to undernourished ewes from day 28 until day 78 of gestation were heavier with more back fat and exhibited hyperglycemia compared to male lambs from dams fed a standard diet throughout pregnancy [22]. In a different study, muscle lipid was increased in lambs from over-nourished ewes, with a higher expression of myostatin and follistatin compared with lambs from the control group [23]. In contrast, lambs from feed restricted ewes at three months of age showed lower muscle lipid content and increased expression of follistatin [23]. In beef cattle, a maternal diet rich in starch was associated with greater intramuscular fat deposition in the progeny [24]. In sheep, birth weight, postnatal fat, and muscle deposition in lambs were altered in response to maternal diets differing in energy sources (alfalfa haylage, limited-corn, and limited distiller grain) supplemented during mid-to late-gestation [25, 26].

Recently, we reported the impact of three maternal diets, alfalfa haylage (HY), limited-corn (CN), or limited distiller grain (DG) at isoenergetic intake from mid- to late-gestation on gene expression of imprinted genes and DNA methyltransferases (*DNMTs*) in four fetal tissues, *longissimus dorsi* (LD) muscle, *semitendinosus* muscle (SM), and subcutaneous and perirenal adipose tissues [27]. In a subsequent study, we assessed the transcriptomic differences in adipose and muscle tissues of fetuses derived from ewes fed each of the HY, CN, and DG diets using RNA-Seq technology [28]. RNA-Seq analysis revealed 823 differentially expressed genes between fetuses of corn- and hay-fed ewes in the LD muscle tissue. Several of these genes are known to be involved in embryonic and fetal development, myogenesis, and muscle differentiation. Other differentially-expressed genes are involved in metabolic processes such as glycolysis, gluconeogenesis, and citrate cycle, with fetal LD samples from hay-fed ewes showing increased expression of genes involved in propionate metabolism and insulin signaling pathways [28]. However, the mechanisms by which different maternal diets alter gene expression and the effects of these diets on the global DNA methylation signatures are not well understood. We hypothesized that different maternal diets during pregnancy will modify the DNA methylation levels in muscle tissues of the fetus. Thus, the objectives of this study were to determine differentially methylated regions (DMRs) in fetal LD muscle tissues from hay- and corn-fed ewes and evaluate the correlation between transcriptomic profiles and DNA methylation signatures.

## Methods

### Ethics statement

This study used previously published data; therefore ethics approval was not required.

### Study design and diets

To study the effects of maternal diet on DNA methylation patterns in fetal LD muscle tissues, mature pregnant Polypay ewes from the Arlington Research Station at the university of Wisconsin-Madison were used in a randomized complete design as described by Lan et al. [27]. In brief, pregnant ewes were randomly divided into two groups which received either a hay or corn diet from mid-gestation (day 67 ± 5) until necropsy (day 131 ± 1). The diets were formulated to meet the nutritional needs of ewes during pregnancy and had equivalent metabolizable energy. To minimize ruminal health problems, haylage was added to the corn diet. Also, concentrations of vitamins and minerals were increased to meet the NRC requirements [29]. Table 1 details the nutrient intake and composition of each diet. A more detailed description of diets can be found in Lan et al. [27] and Radunz et al. [25]. On the day of necropsy, ewes were subjected to anesthesia by intravenous injection of sodium pentobarbital (20 mg/kg) followed by exsanguination of a jugular vein and carotid arteries, as described by Radunz et al. [25]. LD muscle tissues were collected from the fetuses and immediately frozen at − 80 °C for further analysis.

**Table 1:** Maternal diets daily nutrient intake from mid- to late-gestation (modified from Lan et al. [27])

Composition	Hay	Corn
Alfalfa Haylage (kg/day)	2.03	0.14
Corn (kg/day)	–	0.80
Supplement (kg/day)	–	0.23
*Nutrient intake*		
Crude protein (g/days)	383.26	130.63
Methionine (g/days)	6.22	1.47
Serine (g/days)	57.9	8.55
Choline (mg/days)	29.3	7.2
Folate (mg/days)	0.05	6.5

### Whole genome DNA methylation of sheep fetal LD muscle

Genomic DNA was extracted from 16 fetal LD samples, eight per dietary treatment, following a salt-chloroform extraction protocol [30]. DNA concentration and quality were estimated by PicoGreen® (Thermo Fisher, Waltham, MA, USA) and by agarose gel electrophoresis. One μg of genomic DNA was sonicated to produce DNA fragments of about 350 bp. Methyl-binding domain (MBD) enrichment was performed using the MethylMiner™ Methylated DNA Enrichment Kit (Invitrogen, Carlsbad, CA, USA), following the manufacturer’s instructions. Construction of the sequencing library was performed using the TruSeq® Nano Library Preparation Kit (Illumina, San Diego, CA, USA). Libraries were quality checked and quantified on an Agilent 2200 TapeStation, High Sensitivity D1000. The 16 samples were then used for cluster generation and subsequent sequencing in two lanes of an Illumina Hi-Seq 2000 (San Diego, CA), and 100 bp paired-end reads were generated.

### Bioinformatics analysis

Preliminary quality control of raw reads was carried out with FastQC (http://www.bioinformatics.babraham.ac.uk/projects/fastqc/). Illumina raw sequences were filtered with Trimmomatic software v. 0.33 [31] to remove adapters, and a sliding window approach used to remove lower quality bases at the sequence end. Trimmed reads were then aligned to the sheep reference genome Oar_v3.1 using BWA-mem aligner v.0.7.15 [32], and duplicates were marked and removed using Picard-tools v1.107 (http://broadinstitute.github.io/picard). Peak detection was performed for each sample by the ChIPseeqer software [33] using the following parameters: length of reads length equal to 101 bp, and length of the fragments equal to 250 bp. The ChIPseeqer software constructs a read density map by counting the number of overlapping reads at each nucleotide position and then uses a Poisson probability model to compare the observed read count to the expected read count and to compute a normalized peak score for each nucleotide position.

DMRs were detected using the R package DiffBind downloaded from the bioconductor repository (https://bioconductor.org/packages/release/bioc/html/DiffBind.html), which computes differentially bound sites using affinity data. DiffBind works primarily with peak-sets identified by ChIPseeqer and with BAM files containing aligned reads for each sample. To identify binding sites that were differentially bound between diet treatmnet groups, a matrix with the consensus peaks was generated. After setting a contrast between the different conditions, an edgeR algorithm based on an empirical Bayes method [34] method was used to assign a *P*-value and false discovery rate (FDR) to each candidate binding site. The threshold for DMR calling was set to < 0.1. A set of functions from the biomaRt R package (https://bioconductor.org/packages/release/bioc/html/biomaRt.html) was used to query the Ensembl database and annotate the DMRs. The annotation information corresponds to Oar_v3.1, the reference genome used for alignment. Principal component analysis was performed using a function provided by DiffBind to visualize the clustering of the diet-derived samples based on differentially bound sites.

### Validation of the differentially methylated regions (DMRs) using bisulfite sequencing

The DMR located between *PLIN5* and *PLIN4* was chosen for validation because the products of the PLIN genes are involved in coating and packaging functions in the intracellular lipid droplets [35, 36]. *PLIN5* also has a role in lipid metabolism, the coordination of triacylglycerol metabolism, and maintaining insulin action in skeletal muscle [37]. The *LPAR3* and *PLCB4* DMRs were found to be highly methylated in fetal LD samples derived from hay-fed compared with corn fed ewes. In contrast, *ADAMTS12* and the novel lincRNA (ENSOARG00000025638) DMRs were selected because of their high methylation in fetal LD samples from corn-fed compared with hay-fed ewes.

To validate the selected DMRs in the fetal LD muscle tissues, four DNA single-sex pools were created; hay-female (HF, *n* = 4), hay-male (HM, *n* = 4), corn-female (CF, *n* = 3) and corn-male (CM, *n* = 5) (description of pools can be found in Additional file 1: Table S1). Quality and quantity of individual DNA samples used to create the pools were evaluated using Nanodrop ND1000 (Nanodrop Technologies, Montchanin, DE). The Epitect Bisulfite Kit (Qiagen, Germantown, MD) was used for the bisulfite conversion of 500 ng genomic DNA from each pool. Primers specific for bisulfite converted DNA sequences for selected DMRs (Table 2) were designed using the Bisulfite Primers Seeker online tool from Zymosearch (http://www.zymoresearch.de/tools/bisulfite-primer-seeker). The selected DMR regions were amplified by two rounds of PCR (Cycling conditions can be found in Additional file 2: Table S2). Amplification of the whole lincRNA DMR was not possible; therefore, it was divided into two smaller regions. The PCR products were purified using Illustra™ GFX™ PCR DNA and Gel Band Purification kit (GE Healthcare Biosciences, Pittsburgh, PA), ligated into the pGEM-T-easy vector (Promega, Madison, WI), and transformed into KM109 competent cells (Promega) following the manufacturer’s instructions. Colonies were then screened for the presence of the insert by PCR using the same cycling conditions and primers for each DMR. For each pool, 11 to 25 PCR products with the expected fragment size were directly sequenced, and the resulting sequences were aligned with the specific DMR region. The levels of methylation of single CpG sites as well as the overall methylation percentages per pool were assessed using the BISMA online tool [38]. Fisher’s exact test was used to test the significance of the differential methylation between the two diets and sexes.

**Table 2:** Bisulfite converted DNA PCR primers

DMR	Primer Sequence 5′ – 3’	Size (bp)
LPAR3	Forward: AGAGTAAGTAAYGGGTTTAGGTAAAGAG Reverse: ATGAATTCTTCRACACTCAACTTTCTTTATAATCCAA	788
PLIN5-PLIN4	Forward: GGTTTTAGTGGTGGGGTGTTGGGGG Reverse: TCTAACTAACRCAAAATAACTAAAACTCCCAC	520
lincRNA-R2	Forward: TAYGAGGTTAGTTATTATTTGTTTGGATATTTTTAG Reverse: AAAATCTACTAACATAATCCAAAACCATCTTAAC	407
ADAMTS12	Forward: ATAGGGGGGAAAAAAGTAAATAAGTTAGTTGG Reverse: TAATAAAATTCTCTTCTAACTCCCACAAAAC	750
PLCB4	Forward: AAAGGTTTTAGAGGAGAGGGTGAGA Reverse: TTCTCTCACTTTCCAACTCTTCCAATTCTAT	200

### Gene expression analysis of DMR genes

To test the correlation between DNA methylation and gene expression, the selected genes (*LPAR3*, *PLIN4*, *PLIN5, PLCB4*, and *ADAMTS12*) were assessed for differential expression.

Total RNA from the fetal LD muscle samples (*n* = 15) used in the DNA methylation study was extracted using the RNeasy Mini Kit (Qiagen) following the manufacturer’s instructions. RNA purity was assessed using Nanodrop ND1000 (Nanodrop Technologies) and Agilent 2100 Bioanalyzer (Agilent, Santa Clara, CA). A total of 50 ng RNA from each sample was used to synthesize cDNA using the iScript cDNA synthesis Kit (BioRad, Hercules, CA). Primers were designed to span introns using the NCBI Primer-Blast tool (Table 3). The *RPL19* gene was chosen as an endogenous control for its high stability across samples as reported in Lan et al. [27] using the same samples. Gene expression levels were first determined in the HF, HM, CF, and CM cDNA pools by real-time PCR using iTaq SYBR Green kit (BioRad), and then in the individual samples used in pools for the *PLIN4*, *LPAR3*, and *PLCB4* genes. The relative gene expression was evaluated using the 2^-ΔΔCT^ method [39]. Normalized gene expression values (∆CT) of *PLIN4*, *LPAR3,* and *PLCB4* genes from individual samples were used in a general linear mixed model to assess the significance of differential expression, where maternal diet and sex of the fetus were included as fixed effects and dam was a random effect. The analysis was performed using the lme4 package [40] in R [41].

**Table 3:** Relative gene expression, real-time PCR primers

Gene	Primer Sequence 5′ – 3’	Size (bp)
*LPAR3*	Forward: AGGATGTTCAGTTCTTCTCCAC Reverse: GCTTCGTTCCTGTCCACTCA	111
*PLIN4*	Forward: CAGCTGGCTGCTACCCAGCC Reverse: GAGCCTGCTGGGCCTCCTC	209
*PLIN5*	Forward: GGCATGTCAGAAGACGAGGG Reverse: CGCTGTAAGCCTTGGAGACT	141
*RPL19*	Forward: CAACTCCCGCCAGCAGAT Reverse: CCGGGAATGGACAGTCACA	79
*ADAMTS12*	Forward: CCTTGGCTTTCACAGTTGCC Reverse: ATGATGTACAGGTGCCTGCC	103
*PLCB4*	Forward: ACCCTGGTCTGGGAATCCTT Reverse: ATGGCTTGGGTCTGGCTAAA	127

### Whole transcriptome analysis of sheep fetal LD muscle

The transcriptome of fetal LD muscle was evaluated using RNA-Seq as described in detail by Peñagaricano et al. [28]. Briefly, sequence reads were mapped to the reference sheep genome (Oar_v3.1) using Tophat [42]. The resulting alignments were used to reconstruct gene and transcript models using Cufflinks [43]. The companion tool Cuffmerge was used for merging the reference sheep annotation file with individual sample assemblies to combine known annotated transcripts with novel transcripts. Finally, differentially expressed genes between treatments were detected using the software Cuffdiff [44]. RNA-seq and whole genome methylation results were integrated to investigate associations between gene expression and DNA methylation. The correlation between methylation status and gene expression was assessed for active genes located 20 kb up and downstream of DMRs. Intragenic and intergenic methylation correlation with actively transcribed regions were also investigated.

## Results

### Whole genome methylation analysis

**Fig. 1:**
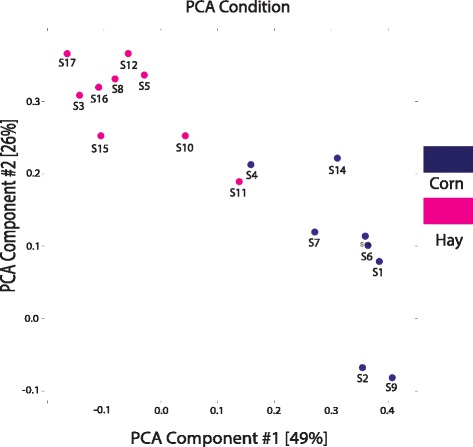
Principal Component Analysis based on whole genome DNA methylation in the sheep fetal LD muscle from hay- and corn-fed ewes

Whole genome methylation analysis (WGMA) revealed 60 DMRs between the fetal LD samples from the two maternal diet groups (Hay vs. Corn). Among these DMRs, 39 had higher methylation levels in the fetal LD muscle from hay-fed dams than corn-fed dams. Of these 39 DMRs, 20 corresponded with 10% FDR, 17 with 5% FDR, and 2 with 2% FDR. The other 21 DMRs had a higher methylation in fetal LD muscle from corn-fed dams. Of these 21 DMRs, 7 corresponded with 10% FDR and 21% corresponded with 5% FDR (list of differentially methylated regions can be found in Additional file 3: Table S3). The DMRs ranged from 232 bp to 1548 bp, with a mean of 663.8 and standard deviation of 236.5 bp. The DMRs were distributed across 19 chromosomes with chromosome 1 having the largest number of DMRs (14/60 or 23.3% of the DMRs). Considering the chromosomal length, chromosome 13 had the highest percentage of DMRs (6.02%) followed by chromosome 6 (5.98%), chromosome 1 (5.08%) and chromosome 7 (4.99%) (The chromosomal distribution can be found in Additional file 4: Figure S1). Principal component analysis (PCA) based on DNA methylation clearly distinguished hay-fed fetal LD samples from corn-fed fetal samples (Fig. 1). Diet type—hay vs. corn—explained 49% of the variance observed in DMRs.

### Validation of differentially methylated regions using bisulfite sequencing

The DMRs in *LPAR3, PLIN5-PLIN4, PLCB4, ADAMTS12*, and the novel lincRNA identified in the whole-genome DNA methylation analysis were selected for validation using the bisulfite-Sanger sequencing method. Methylation levels of the selected DMRs were assessed in the four pools, characterized by sex and diet: HF, HM, CF, and CM.

#### LPAR3 DMR methylation level assessment

**Fig. 2:**
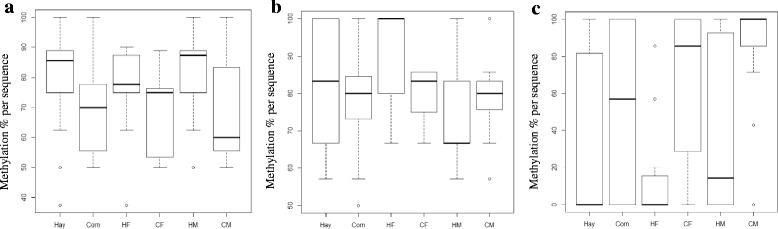
DNA methylation levels in fetal LD muscle from hay- and corn-fed ewes. **a**) *LPAR3* DMR, **b**) *PLIN5-PLIN4* DMR, **c**) lincRNA DMR. **HF**: hay females; **HM**: hay males; **CF**: corn females; **CM**: corn males; **Hay**: male and female pools combined; **Corn**: male and female pools combined. The percentage of DNA methylation per sequence was calculated using the following formula: number of methylated CpG sites / total number of successfully sequenced CpG sites. The BISMA online tool was used to assess the methylation percentage

The *LPAR3* DMR contains 13 CpG sites and is located within the second intron of the gene. A total of 39 sequences from hay pools (17 HF sequences and 22 HM sequences), and a total of 31 sequences from corn pools (16 CF sequences and 15 CM sequences) were analyzed to assess the DNA methylation percentage. The CpG sites 1–3, 12, and 13 were not recognized by the BISMA software and were excluded from the analysis. The percentage of DNA methylation was significantly (*P* = 0.002) higher in fetal LD tissues from hay-fed dams (M + F) with 80.15% methylated CpG sites compared to 69.15% methylation in fetal LD tissues from corn-fed dams (M + F) (Fig. 2a). No significant differential methylation was found between the female pools (77.8% in HF vs. 69.8% in CF); however, the methylation percentage was significantly higher (*P* = 0.027) in HM (83.4%) compared with CM (72.2%).

**Fig. 3:**
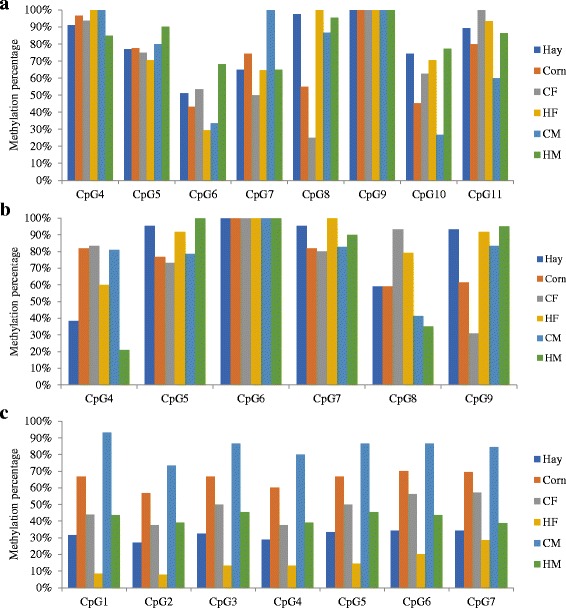
Methylation levels at individual CpG sites. **a**) *LPAR3* DMR, **b**) *PLIN5-PLIN4* DMR, **c**) lincRNA DMR. **HF**: hay females; **HM**: hay males; **CF**: corn females; **CM**: corn males; **Hay**: male and female pools combined; **Corn**: male and female pools combined. The percentage of DNA methylation for each CpG site was calculated using the following formula: number of methylated cytosines at the CpG site/total number of successfully sequenced cytosines at the CpG site under evaluation

Some genes are epigenetically regulated by single CpG sites. Therefore, the methylation levels of each CpG site were analyzed in the fetal LD muscle pools from hay-fed and corn-fed ewes. Significant differences in methylation percentages were found for CpG8, with 97% of CpG sites methylated in fetal LD tissues from hay-fed ewes (M + F) compared to 55% methylation in fetal LD tissues from corn-fed ewe (M + F) (*P* = 1.47e-05). Likewise, CpG10 had significantly (*P* = 0.015) higher methylation in fetal LD samples from hay-fed ewes (74%) compared to fetal LD samples from corn-fed ewe (45%) (Fig. 3a). The methylation of CpG8 was significantly (*P* = 3.32e-06) higher in HF (100%) vs. CF (25%), whereas the methylation of CpG10 was higher (*P* = 0.006) in HM (77%) compared to CM (27%). The methylation of CpG7 was significantly high (*P* = 0.012) in CM (100%) compared to HM (65%) (Fig. 3a).

#### PLIN5-PLIN4 DMR methylation level assessment

The DMR between the *PLIN5* and *PLIN4* was chosen for validation because of the roles of these genes in lipid metabolism and lipid droplets packaging. This DMR contains 9 CpG sites; however, CpGs 1–3 could not be sequenced and therefore were not included in the analysis. A total of 44 sequences from fetuses from hay-fed ewes (24 HF sequences and 20 HM sequences) and 44 sequences from fetuses from corn-fed ewes (15 CF sequences and 29 CM sequences) were analyzed.

The methylation level in the hay diet group (M + F) was 81.89% compared to 78.40% in the corn diet group (M + F), but there was no significant statistical difference between the groups (*P* = 0.68; see Fig. 2b). However, the methylation level in hay-fed females (88.9%) was significantly (*P* = 0.034) higher than that of the corn-fed females (77.6%), while no significant differences were found between the male pools (73.9% in HM vs. 79% in CM).

The analysis of methylation levels of single CpG sites of the PLIN5-PLIN4 DMR (Fig. 3b) showed that CpG5 had significantly (*P* = 0.014) higher methylation in the hay-fed group (95.5%) compared with the corn-fed group (75%). Similarly, CpG9 showed significantly higher methylation in the hay-fed group (93.2%) compared with the corn-fed group (61.3%) (*P* = 0.001), whereas CpG4 was found to be more highly methylated in the corn-fed group (82%) compared with the hay-fed group (38%) (*P* = 0.0004). The methylation level of CpG9 was higher (92%) in the hay-fed female pool compared to corn-fed female pool (31%) (*P* = 0.0002). CpG5 showed significantly higher methylation in the hay-fed male pool (100%) compared with the corn-fed male pool (79%) (*P* = 0.0003). In contrast, CpG4 showed significantly higher methylation in the corn-fed male pool (81%) compared with the hay-fed male pool (21%) (*P* = 0.0003).

#### lincRNA DMR methylation assessment

The lincRNA gene (ENSOARG00000025638) sequence overlaps with the genes *RRP1B*, *HASF2BP*, and *PDXK*. The DMR of this lincRNA is located in the intron of the gene, and contains 17 CpG sites. However, only 7 CpG sites were successfully amplified using DNA bisulfite conversion. For DNA methylation assessment, a total of 38 sequences from the hay-fed group (15 HF sequences and 23 HM sequences) and 31 sequences from the corn-fed pools (16 CF sequences and 15 CM sequences) were analyzed. Methylation differences between the pools are represented in Fig. 2c. Fetal LD samples from corn-fed ewes (M + F) had significantly (*P* = 1.06e-12) higher methylation (65%) compared with fetal LD samples from hay-fed ewes (M + F) (31.6%). Significant differences were also found between the female pools from the different diets (15.2% in HF vs 47.3% in CF) (*P* = 6.76e-07), and between the male pools (42.2% in HM vs 84.5% in CM) (*P* = 6.38e-12) with higher methylation levels in samples from corn-fed ewes. Considering the CpG sites individually, all CpG sites within this DMR were found to be significantly differentially methylated between fetal LD tissues from hay-fed ewes compared with those from corn-fed ewes (*P* < 0.05) (Fig. 3c).

#### ADAMTS12 DMRs methylation assessment

The *ADAMTS12* DMR covers exon 10 and parts of the adjacent introns 9 and 10 of the gene, and it contains 18 CpG sites among which only CpGs 3–17 were successfully sequenced. To evaluate the differential methylation between the pools, 33 sequences from the hay-fed pools (16 HF sequences and 17 HM sequences) and 27 sequences from the corn-fed pools (13 CF sequences and 14 CM sequences) were used. Methylation percentages were 96% in fetal LD samples from hay-fed ewes (M + F) compared to 95.8% in fetal LD samples from corn-fed ewes (M + F) with no significant differences between the groups. Furthermore, no significant differences were found at the single CpG level.

#### PLCB4 DMRs methylation assessment

The *PLCB4* DMR is located in intron 6 of the gene, and contains 3 CpG sites. A total of 29 sequences from hay pools (18 HF sequences and 11 HM sequences) and 35 sequences from corn pools (24 CF sequences and 11 CM sequences) were analyzed for differential DNA methylation. Methylation percentages were not significantly different between the hay and corn-fed pools. Fetal LD samples from hay-fed ewes (M + F) had 94.2% methylation; fetal LD samples from corn-fed ewes (M + F) had 95.89% methylation. Moreover, no significant differences were found between the pools at the single CpG site level.

### Gene expression analysis of the DMR genes

#### Relative gene expression in pools

**Fig. 4:**
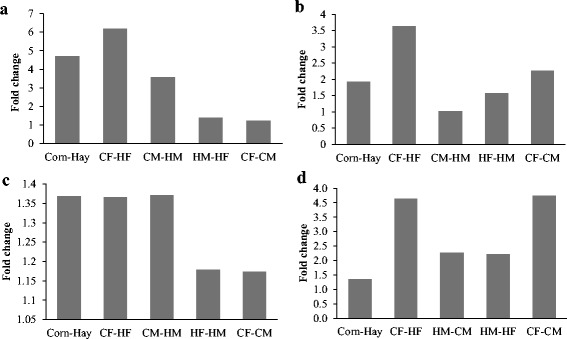
Gene expression profiles of DMRs in the following genes: **a**) *LPAR3*, **b**) *PLIN4*, **c**) *PLIN5* and **d**) *PLCB4*. The fold difference in expression was calculated using the 2^-∆∆CT^ method. **Corn**: male and female pools combined; **Hay**: male and female pools combined; **CF**: corn females; **HF**: hay females; **CM**: corn males; **HM**: hay males

The gene expression profiles of the DMR genes *LPAR3, PLIN4, PLIN5, and PLCB4* were calculated as a fold change (FC) between the different pools using the 2^-∆∆CT^ method (Fig. 4). *ADAMTS12* was not found to be expressed in LD muscle. The highest fold changes in gene expression in fetal LD muscle were found for *LPAR3* between corn-fed and hay-fed groups (Corn- Hay) with FC = 4.7, the female pools (CF-HF) with FC = 6.2, and the male pools (CM-HM) with FC = 3.57 suggesting a higher expression in the fetal LD pools from corn-fed ewes (Fig. 4a).

For *PLIN4,* gene expression was higher in the fetal LD tissues from corn-fed ewes than those from hay-fed ewes (FC = 1.92). Differences in expression were observed for CF-HF (FC = 3.63) with higher expression in the corn-fed female pool. Likewise, a higher level of expression of this gene was observed in the female pool (CF) compared to the male pool (CM) within the corn-fed group (FC = 2.26) (Fig. 4b). For the *PLIN5* gene, all FCs among fetal LD tissues were < 1.4 (Fig. 4c).

Expression of *PLCB4* was higher in corn female pools compared to corn female pools (FC = 4.14). Differences in expression were also observed in female and male pools from the corn-fed ewes (CF-CM) (FC = 4.25) with higher levels of expression in the female pool. Likewise, *PLCB4* had a higher expression in the hay-fed male pool compared with the corn-fed male pool (HM-CM) (FC = 2.27). In addition, the hay-fed male pool had higher expression compared with the hay-fed female pool (FC = 2.21) (Fig. 4d).

#### Relative gene expression in individual samples

**Fig. 5:**
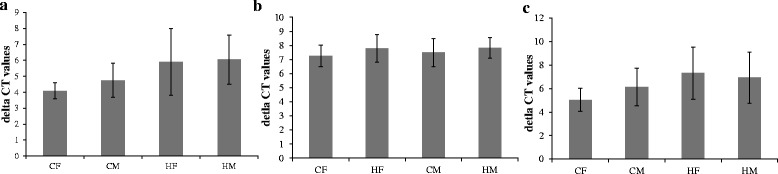
Normalized gene expression values: **a**) *LPAR3*, **b**) *PLIN4* and **c**) *PLCB4*. **CF**: corn females; **CM**: corn males; **HF**: hay females; **HM**: hay males. ∆CT (CT_Target gene_ – CT_reference gene ‘RPL19’)_ values for individual samples were averaged. Error bars represent the standard error

To validate the results of gene expression in pools, expression of the *LPAR3*, *PLIN4*, and *PLCB4* genes was analyzed in individuals from which the pools were composed, using a linear mixed model. Normalized gene expression values (∆CT) for each gene are shown in Fig. 5. The effect of diet on the expression of *LPAR3* gene was significant (*P* = 0.03) with a higher expression in fetal LD samples from corn-fed ewes (Fig. 5a), while the sex of the fetus had no significant effect (*P* = 0.61). Neither diet nor sex had a significant effect on the expression of *PLIN4* (*P* = 0.15 and *P* = 0.49, respectively) (Fig. 5b) or *PLCB4* (*P* = 0.21 and *P* = 0.75, respectively) (Fig. 5c).

### Comparison of whole genome DNA methylation and RNA-Seq data

A total of 29 out of the 60 DMRs were located within or near (± 20 kb) 49 annotated transcribed regions in the sheep genome. Thirty-three of these genes showed active expression in fetal LD muscle (list of the differentially methylated regions and the associated 33 genes can be found in Additional file 5: Table S4). Methylation levels in these regions were negatively correlated (r = 0.18) with gene expression levels. Interestingly, 13 genes harboring DMRs showed differential expression (*P* < 0.10) between maternal diets. Furthermore, DMRs within the gene body of active genes were shown to be associated with differential expression of neighboring genes. Notably, higher intra-gene DNA methylation was associated with higher gene expression, while higher levels of DNA methylation near but outside the gene were mostly associated with lower gene expression.

## Discussion

In a previous study, we reported that maternal diet during the last trimester of pregnancy can alter the expression and DNA methylation of imprinted genes and DNMTs in different fetal tissues [27]. In a subsequent study, transcriptomic analysis of fetal LD muscle tissue revealed many differentially expressed genes between fetuses from mothers that were fed hay or corn diets; many of those genes are directly involved in fetal development (e.g., *ANKRD11*, *AX1N1*, *EPN1*, *MBD3,* and *WDTC1*) and myogenesis (e.g., *ANKRD1*, *BCL9L*, *HIRA*, *MYH13*, *SYNPO2L,* and *MYOD1*) [28]. The results of these studies indicate that maternal diets can alter the gene expression profile in the offspring. Therefore, to better understand the mechanisms underlying the observed differential expression in the LD muscle, we investigated the global methylation patterns in fetuses from ewes fed one of two diets and their association with gene expression. Many DMRs including single CpG sites were found to be different between the two groups of fetal tissues. In addition, integrative analysis of whole-genome DNA methylation and transcriptomic data revealed a remarkable overlap between DMRs and differential gene expression profiles.

### Whole-genome DNA methylation patterns differ between fetuses from hay- and corn-fed dams

Most studies investigating the effect of maternal nutrition on DNA methylation in livestock have focused on candidate genes involved in metabolic pathways and growth [20, 21, 27, 45]. This study reports the impact of different maternal diets during gestation on the whole genome methylation patterns including DMRs in the fetus. Differentially methylated loci between the two maternal diets were characterized using the methyl binding domain-based (MBD) protein which has affinity to methylated CpG sites [46]. A total of 60 DMRs between the two diet groups were identified in which 39 DMRs were highly methylated in fetal LD samples from hay-fed ewes compared to 21 DMRs in fetal LD samples from corn-fed ewes. 49% of DNA methylation differences were explained by diet. Fetuses from the maternal hay diet had higher levels of nutrients known to be involved in the one-carbon cycle where the methyl group donor (SAM) for DNA methylation is produced, which may explain the large number of methylated regions in fetuses derived from hay-fed dams. Choline and folate are converted to betaine and 5-methyltetrahydrofolate (5MTHF), respectively. Both betaine and 5MTHF donate a methyl group to homocysteine resulting in its conversion to methionine [14, 16]. The methyl group donor SAM is then produced from methionine [47] and is used by the DNMTs for DNA methylation during critical periods of fetal development.

Among genes associated with significant DMRs identified here some are involved in lipid metabolism, muscle growth, and insulin signaling such as *LPAR3*, *PLIN4*, *PLIN5*, *NBEA*, and *SREBP1*.The lysophosphatidic acid receptor 3 (LPAR3), a member of G protein-coupled receptor binding lysophosphatidic acid molecules, is involved in major signaling pathways affecting embryogenesis, nervous system development, vascular development, uterine implantation, immune cell trafficking, and inflammatory reactions [48–50]. In skeletal muscle, the LPA receptor is thought to be involved in the induction of muscle hypertrophy, metabolism, and regeneration through coupling with Gαi a G-protein [51] and is required for skeletal muscle growth and satellite cell proliferation and differentiation [52]. This suggests that maternal diet may affect developmental processes in the fetus.

PLIN4 and PLIN5, members of the perilipin family, are involved in intracellular lipid droplets coating and packaging [35, 36]. PLIN5 plays an important role in triacylglycerol metabolism and insulin action in skeletal muscle, and PLIN5-null mice developed skeletal muscle insulin resistance [37]. Neurobeachin (NBEA), a regulator of synaptic protein targeting, has been reported to affect body weight due to increase adipose tissue mass in haploinsufficient mice [53]. *Sterol regulatory element-binding protein 1* (SREBP-1) belongs to a family of transcription factors known to be highly expressed in adipose tissues regulating several metabolic processes such as fatty acid synthesis, protein synthesis, and cholesterol metabolism. Thus, maternal diet is also likely to affect fat metabolism in the developing fetus. Overexpression of SREBP-1 in muscle induces atrophy and downregulation of the expression of *MYOD1*, *MYOG* and *MEF2C* [54, 55]. ADAMTS12 is a member of disintegrin and metalloproteinase with thrombospondin motifs family which is important in the turnover of extracellular matrix proteins in various tissues [56]. In contrast, PLCB4, a phospholipase Cβ, catalyzes the formation of 1,4,5-triphosphate and diacylglycerol form phosphatidylinositol 4,5-bisphosphate [57, 58] and is involved in signaling pathways such as calcium signaling pathway [57] as well as lipid metabolism [58, 59] and the novel lincRNA have no clear functions in skeletal muscle and warrant further investigation.

Unlike most of the studies in livestock which have assessed the effects of over- or under-nutrition on the epigenomes and phenotypes of offspring, this study compared the effects of two maternal diets with differing *sources* of energy, while maintaining the nutrient requirements of the National Research Council for sheep [29]. Overall, the results suggest that maternal diet during pregnancy has a global effect on DNA methylation patterns in sheep fetal LD muscle and in particular on genes involved in lipid metabolism, muscle growth, and insulin resistance.

### Validation of significant DMRs using bisulfite sequencing

The DMRs of *LPAR3*, *PLIN5-PLIN4*, *PLCB4*, *ADAMTS12*, and a novel lincRNA (ENSOARG00000025638) were selected for validation by bisulfite sequencing. Selection criteria were based on DMR location (inter/intragenic) and possible function of the gene in muscle tissue such as muscle growth, lipid metabolism, and insulin resistance. Methylation percentages were calculated for the overall DMR as well as at single CpG sites level for each constructed pool (HF, HM, CF, CM).

Bisulfite sequencing analysis of the *LPAR3* DMR showed an overall higher methylation percentage in fetuses from hay-fed vs. corn-fed ewes, thus confirming the whole genome methylation results. However, although significant differences were found between male pools (HM vs. CM), no differences were found between female pools (HF vs. CF). For the *PLIN5-PLIN4* DMR, significant DNA methylation differences were found between female pools but not between male pools. DNA methylation analysis of the lincRNA DMR revealed high methylation levels in corn fetuses compared to hay fetuses confirming the whole genome methylation results. Significant differences were also found between the female pools, and between the male pools with higher methylation levels in fetuses from corn-fed ewes. These findings suggest that effects of maternal diet are gene- and sex-specific. The results presented here are consistent with previous studies demonstrating sexual dimorphism in response to maternal diet. Recently, we reported that imprinted genes and DNMTs including *DNMT1*, *DNMT3a*, and *DNMT3b* showed sex- and tissue-specific DNA methylation and expression patterns in sheep fetuses in response to different maternal diets during pregnancy [27]. Gallou-Kabani et al. [60] have also reported that sexual dimorphism in terms of gene expression and DNA methylation of imprinted genes in placenta was associeted with a high-fat diet of pregnant ewes. The sexual dimorphism observed in this study in response to maternal diet during the last trimester of pregnancy could alter fetal programming of male vs. female fetuses and hence affect postnatal traits of the offspring. The DMRs of *ADAMTS12* and *PLCB4* were both highly methylated in all pools with no significant methylation differences between the diets. This discrepancy between whole-genome methylation results and the bisulfite sequencing can be explained by the different methods used to assess methylation levels. When using bisulfite conversion, 5-hmC is indistinguishable from 5-mC thus CpG sites identified as methylated may be hydroxymethylated [61], whereas the methyl binding domain protein (used in the whole-genome methylation) binds specifically to methylated cytosine in the form of 5mC and not 5hmC [62, 63].

Significant methylation differences were found between fetal LD samples from hay- and corn-fed ewes for the *LPAR3* and *PLIN5-PLIN4* DMRs at the level of single CpG sites. Indeed, single CpG site methylation has been reported to control gene expression, e.g., the binding of Oct-1 was inhibited by methylation of a single CpG site in the human *IL2* promoter resulting in decreased transcription of the gene [64]. Similarly, a cluster of methylated CpG sites located in the promoter region resulted in gene silencing of *XAF1* [65]. In fish, it was found that methylation of a single CpG site within exon 8 of the *GHR1* decreased transcription of the gene [66]. Likewise, hypermethylation of one intronic CpG dinucleotide was associated with the loss of *PMP24* expression [67]. Also, a single CpG site methylation was found to down-regulate the expression of *METTL7A* in thyroid cancer [68]. Thus, the differential single CpG methylation observed in this study may be involved in modulating gene expression in the fetal tissues.

### The correlation between DNA methylation and expression of the validated DMR genes

DMRs play a major role in transcriptional regulation and can be associated with either gene silencing or transcription elongation, depending upon the specific location of the methylated CpG sites in the gene [69]. Although a negative correlation between DNA methylation in the promoter regions and gene expression is well-established, both negative and positive correlations between gene-body methylation and expression have been reported. In this study, the introic DMR of *LPAR3* showed higher methylation in hay fetsues compared with corn fetuses whereas expression of *LPAR3* was lower in the hay fetuses compared with corn fetuses. In contrast, Lan et al. [27] found that methylation of the DMR located in intron 2 of *IGF2R* was positively associated with expression of the gene. Similarly, in a colon cancer study, gene expression was positively correlated with gene-body methylation [70]. Furthermore, gene-body demethylation has been associated with downregulation of genes, thus demonstrating a causative relationship between DNA methylation and gene expression [70]. In mice, folic acid supplementation during pregnancy led to hypermethylation of gene bodies, which in turn was associated with both up- and down-regulation of genes in the cerebral hemispheres of the offspring [71]. Thus, these observations underline the complexity of the relationship between gene-body methylation and gene expression.

The intergenic DMR between *PLIN5* and *PLIN4* showed significant methylation differences between female pools (HF vs. CF) with higher methylation in the hay fetuses. While *PLIN5* showed no expression differences among the fetuses from both diets, or sexes, *PLIN4* was highly expressed in the corn fetuses compared to the hay fetuses. In addition, a differential methylation between female pools from both diets was found at the single CpG site level of the DMR. Although, a causative relationship between the observed pattern of DNA methylation and *PLIN4* expression needs to be examined, the observed negative correlation suggests this DMR as potential regulatory region of the *PLIN4* gene. Indeed, 79 bp in the sheep DMR are highly conserved with the enhancer region located between *PLIN5* and *PLIN4* in the human, pig, and cattle genomes. Thus, although a direct relationship between the pattern of DNA methylation in this DMR and *PLIN4* expression was not studied, the observed negative correlation and the similarity to the human enhancer region suggest that this locus is a potential regulatory region.

The *ADAMTS12* DMR had high levels of methylation with no significant differences between the fetuses, and the gene was found to be not expressed in the fetal tissues. The DMR of this gene covers the entire region of exon 10 and regions of the neighboring introns, and Methprimer suggested that it contains a predicted CpG island (Additional file 6: Figure S2) [72]. Generally, CpG islands overlap with regulatory sequences, such as promoters or enhancers that regulate gene expression [73]. Also, methylation of CpG islands found within regulatory regions is associated with gene repression by blocking binding of regulatory proteins [7, 74], recruitment of histone deacetylases, and formation of inactive chromatin [3]. Thus, the methylation of the *ADAMTS12* DMR may suppress expression, although this assumption needs additional investigation. The bisulfite sequencing of the *PLCB4* DMR was not validated in this study, although remarkable gene expression differences were observed between male and female fetuses. Furthermore, the mixed model found no significant effect of the maternal diet on the differential expression of this gene. These findings show that the studied DMR does not correlate with expression of this gene. Therefore, it is possible that the transcription of *PLCB4* is regulated by mechanisms other than DNA methylation at this location.

The function of the novel lincRNA (ENSOARG00000025638) is not known and its expression profile was not determined in the present study. However the differential methylation between hay and corn fetuses was highly significant. Given that the DMR of the lincRNA overlaps with the *RRP1B*, *HASF2BP*, and *PDXK* genes, the differential methylation found in the fetuses in response to the maternal diet may play a role in the regulation of the three overlapping genes.

### Comparative analysis RNA-Seq and whole genome methylation data

A total of 29 DMRs that were located within or near 49 annotated transcribed regions in the sheep genome were associated with 13 transcripts found to be differentially expressed in the RNA-Seq analysis. Comparative analysis of RNA-Seq and whole genome methylation data showed that gene body methylation was positively associated with gene expression, whereas intergenic DNA methylation, known to harbor promoters and enhancer regions, was negatively correlated with gene expression. A study in precursor β cells revealed that transcription factors and enhancers bind to intergenic regions and that hypermethylation in these regions was found to be associated with downregulation of neighboring genes [75].

In the present study, the *PDXK*, *PLIN5* and *PLIN4* genes showed a correlation between differential expression and DNA methylation of intra- and inter-genic DMRs. *PDXK* is a pyridoxal kinase, which has a role in converting vitamin B6 into its active form, phosphorylated pyridoxal [76], which is needed for the conversion of THF into methylene THF which in turn is either converted into thymidine or to 5MTHF used in methionine production [77]. Interestingly, *PDXK* expression was found to be correlated with DNA methylation pattern of the lincRNA (ENSOARG00000025638), which was validated by bisulfite sequencing. These findings suggest that DNA methylation in this genomic region modulates the expression of *PDXK* which in turn could affect methionine and thymidine production and potentially influence the one carbon metabolism and hence genome-wide methylation patterns.

The two perilipin genes *PLIN4* and *PLIN5* are known to be involved in lipid droplet coating and lipid metabolism, with *PLIN5* playing a role in insulin resistance. These two genes could be important in lipid deposition in skeletal muscle and insulin resistance later in life [35, 36]. The correlation was seen between expression and DNA methylation of *PLIN4* in the female pools, whereas the expression of *PLIN5* was not significantly different between the diet groups. In contrast, comparative analysis showed that *PLIN5* is differentially expressed. This discrepancy could be due to the pooling strategy used in validating the whole genome methylation data and expression profile for this gene or due to the sensitivity of RNA-Seq compared to real-time PCR used to assess the expression level of *PLIN5*. Overall, the comparative analysis of whole genome DNA methylation and transcriptomics provides a tool to characterize regulation mechanisms for genes influenced by maternal nutrition and contribute to a better understanding of the mechanisms underlying phenotypic variation.

One limitation of the present study is that the nature of the ruminant digestive system and its microbiota makes it difficult to assess the amounts of and type of nutrients provided to the fetus during pregnancy. This limitation could be overcome in future studies by supplementing maternal diets with rumen-protected nutrients. Nutrient partitioning during development between twins may differ and could influence the observed DNA methylation patterns in the fetuses. In this study the DNA pools were balanced in terms of the number of twins and singletons for each dietary group, however future studies should focus only on singleton pregnancies.

## Conclusions

The present study reports the effects of maternal diets differing in energy sources supplemented during later pregnancy on whole genome methylation and transcriptomic patterns in the offspring of sheep. In response to maternal diet during pregnancy, differential DNA methylation was observed in fetal muscle tissue, and this methylation was associated with differential gene expression. Three DMRs were validated using bisulfite sequencing in which two DMRs were found to be associated with expression of genes involved in metabolism and insulin resistance, providing initial data for further studies on regulatory mechanisms and the effect of DNA methylation on gene expression.

## Additional files

Additional file 1: Table S1. Description of DNA pools with number of fetuses and their respective dams. (DOCX 14 kb)

Additional file 2: Table S2. Bisulfite converted DNA PCR conditions. (DOCX 15 kb)

Additional file 3: Table S3. List of Differentially methylated regions between hay and corn fetuses. (XLSX 17 kb)

Additional file 4: Figure S1. Chromosomal distribution of differentially methylated regions between hay and corn fetuses. (PPTX 62 kb)

Additional file 5: Table S4. List of differentially methylated regions with associated actively transcribed regions. (DOCX 18 kb)

Additional file 6: Figure S2. ADAMTS12 predicted CpG island using Methprimer. (PPTX 71 kb)
