# Supplementary material for: Integrative analysis of methylomic and transcriptomic data in fetal sheep muscle tissues in response to maternal diet during pregnancy
Source: BMC Genomics. 2018 Feb 6;19:123. doi: 10.1186/s12864-018-4509-0 (PMC5801776; doi:10.1186/s12864-018-4509-0)
Supplement: Supplementary file 1 — Description of DNA pools with number of fetuses and their respective dams. (DOCX 14 kb) [file 12864_2018_4509_MOESM1_ESM.docx]

**Additional file 1: TableS1.** Description of DNA pools with number of fetuses and their respective dams

| **Alfalfa Haylage** | | **Limit-fed** | |
| --- | --- | --- | --- |
| ***Females pool (HF)*** | ***Males pool (HM)*** | ***Females pool (CF)*** | ***Males Pool (CM)*** |
| 6385 A  7343 A  7343 B  8330 A | 6385 B  7321 A  7321 B  8330 B | 5393 A  6379 A  8458 A | 5393 B  7307 A  7307 B  8382 A  8382 B |

Sixteen samples of fetal longissimus dorsi were collected.

Numbers represent dams and letters represent offspring. A and B means twins from a given dam.
