# Supplementary material for: Integrative analysis of methylomic and transcriptomic data in fetal sheep muscle tissues in response to maternal diet during pregnancy
Source: BMC Genomics. 2018 Feb 6;19:123. doi: 10.1186/s12864-018-4509-0 (PMC5801776; doi:10.1186/s12864-018-4509-0)
Supplement: Supplementary file 2 — Bisulfite converted DNA PCR conditions. (DOCX 15 kb) [file 12864_2018_4509_MOESM2_ESM.docx]

**Additional file 2. Table S2**. Bisulfite converted DNA PCR conditions

| DMR | Cycling Condition |
| --- | --- |
| PLINs,  LPAR3,  PLCB4 | Initial Denaturation: 95°C for 5 minutes  Denaturation: 95°C for 30 seconds  Annealing: 66°C for 30 seconds  Extension: 72°C for 30 seconds  35 cycles  Final Extension: 72°C for 10 minutes |
| lincRNA | Initial Denaturation: 95°C for 2 minutes  Denaturation: 95°C for 30 seconds  Annealing: 55.8°C for 30 seconds  Extension: 68°C for 30 seconds  35 cycles  Final Extension: 68°C for 5 minutes |
| ADAMTS12 | Initial Denaturation: 94°C for 4 minutes  Denaturation: 94°C for 30 seconds  Annealing: 59°C for 30 seconds  Extension: 72°C for 45 seconds  40 cycles  Final Extension: 72°C for 5 minutes |
