# Supplementary figures and images for: Integrative analysis of methylomic and transcriptomic data in fetal sheep muscle tissues in response to maternal diet during pregnancy
Source: BMC Genomics. 2018 Feb 6;19:123. doi: 10.1186/s12864-018-4509-0 (PMC5801776; doi:10.1186/s12864-018-4509-0)

## Slide 1
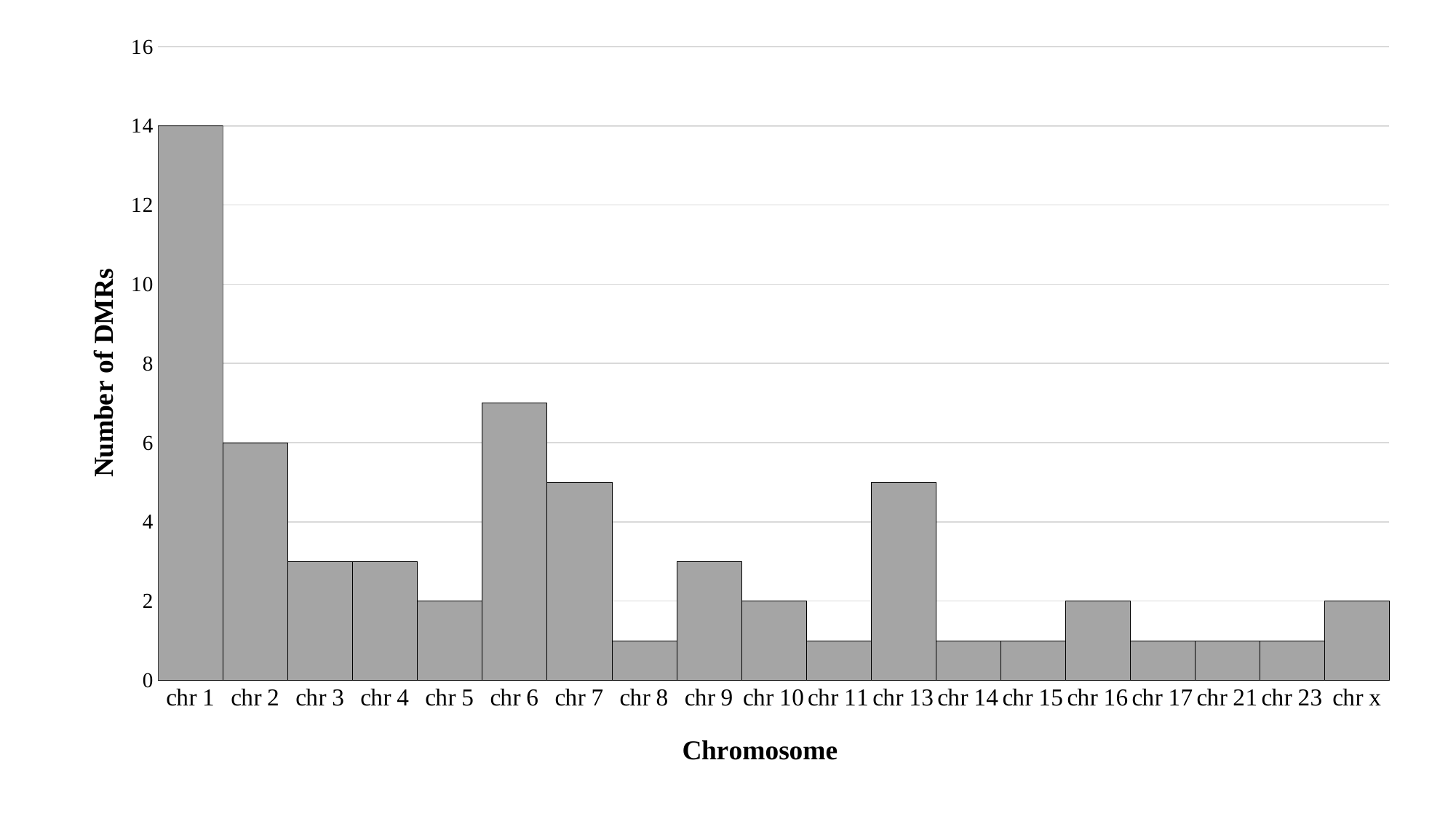

### Chart
| Category | |
|---|---|
| chr 1 | 14.0 |
| chr 2 | 6.0 |
| chr 3 | 3.0 |
| chr 4 | 3.0 |
| chr 5 | 2.0 |
| chr 6 | 7.0 |
| chr 7 | 5.0 |
| chr 8 | 1.0 |
| chr 9 | 3.0 |
| chr 10 | 2.0 |
| chr 11 | 1.0 |
| chr 13 | 5.0 |
| chr 14 | 1.0 |
| chr 15 | 1.0 |
| chr 16 | 2.0 |
| chr 17 | 1.0 |
| chr 21 | 1.0 |
| chr 23 | 1.0 |
| chr x | 2.0 |

Supplement: Supplementary file 4 — Chromosomal distribution of differentially methylated regions between hay and corn fetuses. (PPTX 62 kb) [file 12864_2018_4509_MOESM4_ESM.pptx]

## Slide 1
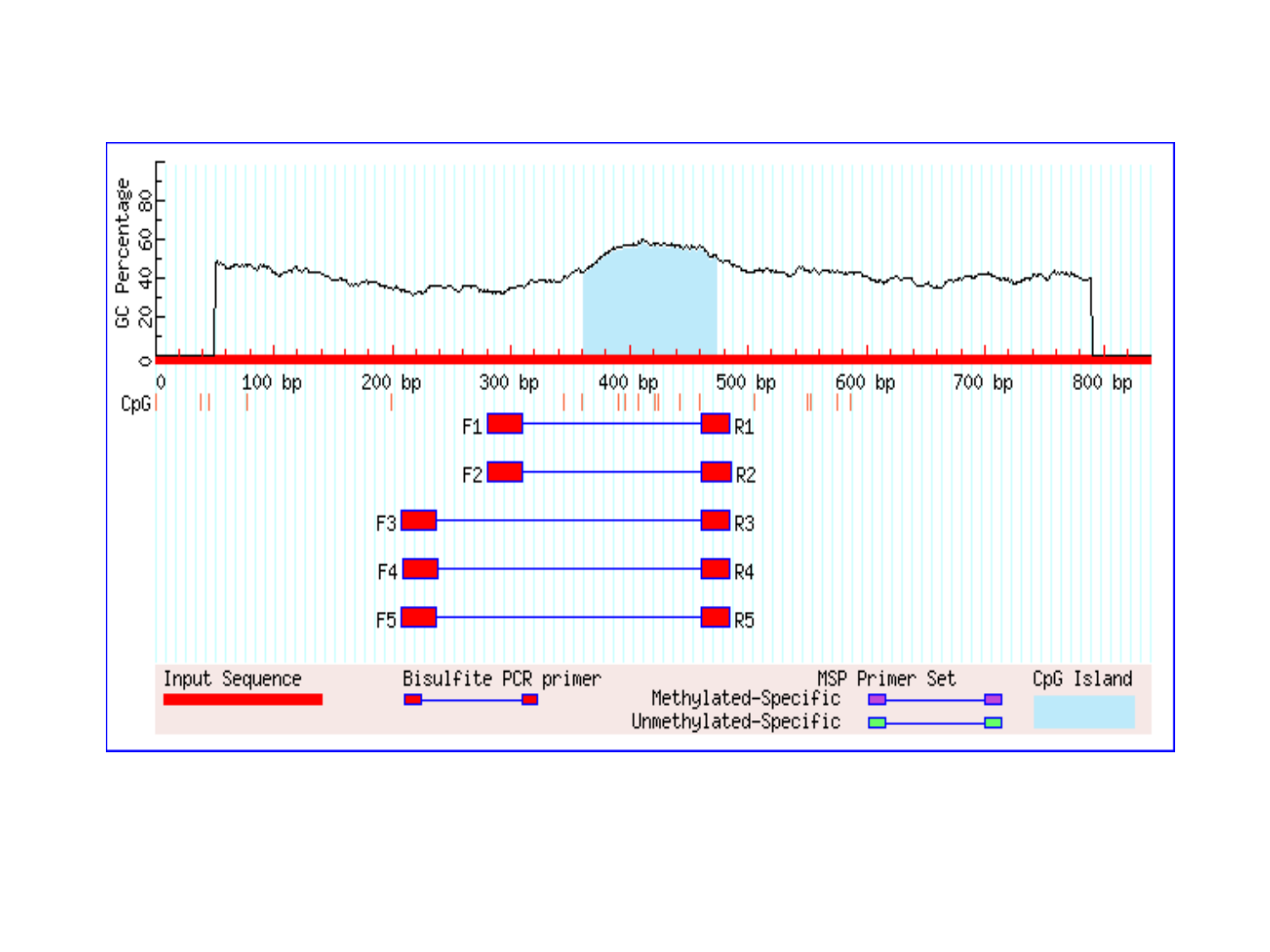

Supplement: Supplementary file 6 — ADAMTS12 predicted CpG island using Methprimer. (PPTX 71 kb) [file 12864_2018_4509_MOESM6_ESM.pptx]
