# Supplementary material for: Integrative analysis of methylomic and transcriptomic data in fetal sheep muscle tissues in response to maternal diet during pregnancy
Source: BMC Genomics. 2018 Feb 6;19:123. doi: 10.1186/s12864-018-4509-0 (PMC5801776; doi:10.1186/s12864-018-4509-0)
Supplement: Supplementary file 5 — List of differentially methylated regions with associated actively transcribed regions. (DOCX 18 kb) [file 12864_2018_4509_MOESM5_ESM.docx]

**Additional file 5. Table S4.** List of differentially methylated regions with associated actively transcribed regions.

| **DMR** | **Active Genes** | **Location** |
| --- | --- | --- |
| chr1:224617451-224618164 | ENSOARG00000001927 | Intergenic |
| chr1:224617451-224618164 | ENSOARG00000001927,  ENSOARG00000014958 | Intergenic |
| chr11:5783217-5783701 | PCTP | Intergenic |
| chr13:36148708-36149291 | RAB18 | Intergenic |
| chr13:62253699-62254466 | CDK5RAP1,SNTA1 | Intergenic |
| chr13:62253699-62254466 | NA | Intergenic |
| chr13:62253699-62254466 | NA | Intergenic |
| chr5:17188921-17189308 | LRG1 | Intergenic |
| chr5:17188921-17189308 | PLIN5 | Intergenic |
| chr5:17188921-17189308 | PLIN4 | Intergenic |
| chr5:17188921-17189308 | ENSOARG00000009468 | Intergenic |
| chr6:84940340-84940940 | SULT1B1 | Intergenic |
| chr1:243358666-243359415 | SLC9A9 | Intragenic |
| chr1:244196373-244196630 | PCOLCE2 | Intragenic |
| chr1:272395666-272396436 | PLCL2 | Intragenic (TBC1D5) |
| chr1:272395666-272396436 | TBC1D5 | Intragenic |
| chr1:42682008-42682542 | SERBP1 | Intragenic |
| chr1:42682008-42682542 | NA | Intragenic (SERBP1) |
| chr1:42682008-42682542 | NA | Intragenic (SERBP1) |
| chr10:24943966-24944693 | SUPT20H | Intragenic (ALG5) |
| chr10:24943966-24944693 | ALG5 | Intragenic |
| chr10:24943966-24944693 | EXOSC8 | Intragenic (ALG5) |
| chr10:26047624-26048353 | NBEA | Intragenic |
| chr13:1832872-1833103 | PLCB4 | Intragenic |
| chr16:40036423-40037261 | ADAMTS12 | Intragenic |
| chr2:36256878-36257657 | FRMD3 | Intragenic |
| chr2:86639975-86640322 | ADAMTSL1 | Intragenic |
| chr4:30918087-30918838 | RAPGEF5 | Intragenic |
| chr4:86542263-86543087 | AASS | Intragenic |
| chr6:45988464-45989245 | TBC1D19 | Intragenic |
| chr10:26047624-26048353 | MAB21L1 | Intragenic (NBEA) |
| chr1:261862656-261863385 | PDXK | Intragenic (lincRNA ENSOARG00000025638) |
